# Designing a B Cell-Based Vaccine against a Highly Variable Hepatitis C Virus

**DOI:** 10.3389/fmicb.2017.02692

**Published:** 2018-01-15

**Authors:** Thomas R. Fuerst, Brian G. Pierce, Zhen-Yong Keck, Steven K. H. Foung

**Affiliations:** ^1^Institute for Bioscience and Biotechnology Research, University of Maryland, Rockville, MD, United States; ^2^Department of Cell Biology and Molecular Genetics, University of Maryland, College Park, MD, United States; ^3^Department of Pathology, Stanford University School of Medicine, Stanford, CA, United States

**Keywords:** hepatitis C virus, vaccine design, epitopes, virus neutralization, antigenic domains, human monoclonal antibodies

## Abstract

The ability to use structure-based design and engineering to control the molecular shape and reactivity of an immunogen to induce protective responses shows great promise, along with corresponding advancements in vaccine testing and evaluation systems. We describe in this review new paradigms for the development of a B cell-based HCV vaccine. Advances in test systems to measure *in vitro* and *in vivo* antibody-mediated virus neutralization include retroviral pseudotype particles expressing HCV E1E2 glycoproteins (HCVpp), infectious cell culture-derived HCV virions (HCVcc), and surrogate animal models mimicking acute HCV infection. Their applications have established the role of broadly neutralizing antibodies to control HCV infection. However, the virus has immunogenic regions in the viral envelope glycoproteins that are associated with viral escape or non-neutralizing antibodies. These regions serve as immunologic decoys that divert the antibody response from less prominent conserved regions mediating virus neutralization. This review outlines the immunogenic regions on E2, which are roughly segregated into the hypervariable region 1 (HVR1), and five clusters of overlapping epitopes designated as antigenic domains A-E. Understanding the molecular architecture of conserved neutralizing epitopes within these antigenic domains, and how other antigenic regions or decoys deflect the immune response from these conserved regions will provide a roadmap for the rational design of an HCV vaccine.

## Introduction

Chronic hepatitis C virus (HCV) infection often leads to chronic hepatitis, liver failure and hepatocellular carcinoma (Mohd Hanafiah et al., 2013). The virus has infected 3% of the global population with an annual rate of 3–4 million new infections. The number of deaths associated with HCV infection in the United States has been increasing, and it is the primary indication for liver transplantation in the Western world (Rosen, 2011; Ly et al., 2016). While advances in HCV treatment with direct-acting antivirals (DAA) have led to cure rates over 90% with HCV treatment, high costs limit access to these drugs in developing and in developed countries (Cox and Thomas, 2013; Callaway, 2014; Chung and Baumert, 2014). Diagnosis of HCV infection often occurs at a late stage and successful DAA treatment will not alter the risk for cancer. DAA treatment is also less successful with genotype 3 infection, decompensated liver disease and transplant recipients. In addition, reinfection remains a problem even after successful treatment in subjects with continued at risk behavior such as injection drug use. For these reasons, an effective prophylactic vaccine is needed.

The genetic diversity of HCV of at least seven HCV genotypes that differ up to 30% in nucleotide sequence, which can be further subdivided into 67 subtypes (Tarr et al., 2015), poses a major challenge to develop a pan-genotypic vaccine (Walker, 2017). Another hurdle is that immune correlates of protection have yet to be defined for HCV. Nonetheless, B cell immunity contributes to the host defense against HCV infection, although it is more complex than sterilizing immunity as observed for hepatitis A, B and E vaccines (Walker, 2017). During acute infection, a robust neutralizing antibody response correlates with spontaneous resolution of infection (Saito et al., 1990; Lavillette et al., 2005; Pestka et al., 2007; Dowd et al., 2009; Lawitz et al., 2013; Osburn et al., 2014; Walker and Grakoui, 2015). Passive immunization with anti-HCV antibodies before HCV challenge prevented infection in animal models (Farci et al., 1996; Law et al., 2008; Dorner et al., 2011; Morin et al., 2012; Bukh et al., 2015). However, passive immunization of chimpanzees with antibodies that neutralized infectivity of several HCV genotypes in cell culture only delayed infection with homologous virus challenge and failed to protect against heterologous virus strains (Bukh et al., 2015). Other complicating factors for vaccine development include viral envelope proteins of low immunogenicity as suggested by a slow antibody response during acute infection (Logvinoff et al., 2004; Dowd et al., 2009; Liang, 2013) and antibody responses directed at regions of the viral envelope that have a high mutational rate of change (Weiner et al., 1992; Shimizu et al., 1994). In addition, antibody responses in an infected individual tend to lag behind the contemporaneous strains of virus in circulation (von Hahn et al., 2007).

Making it more challenging, accessibility to specific antigenic regions and the induction of neutralizing antibodies to these regions can be hindered by glycan shielding (Helle et al., 2007, 2010). Moreover, direct cell-to-cell transmission of the virus, circulating virions in complex with lipoproteins and downregulation of major histocompatibility complex (MHC) expression are other mechanisms for the virus to escape protective immunity (For review see, André et al., 2002; Cashman et al., 2014; Dunlop et al., 2015; Pierce et al., 2016a). Development of an effective vaccine for HCV must consider these factors.

## Challenges of antigenically variable viruses

**Figure 1:**
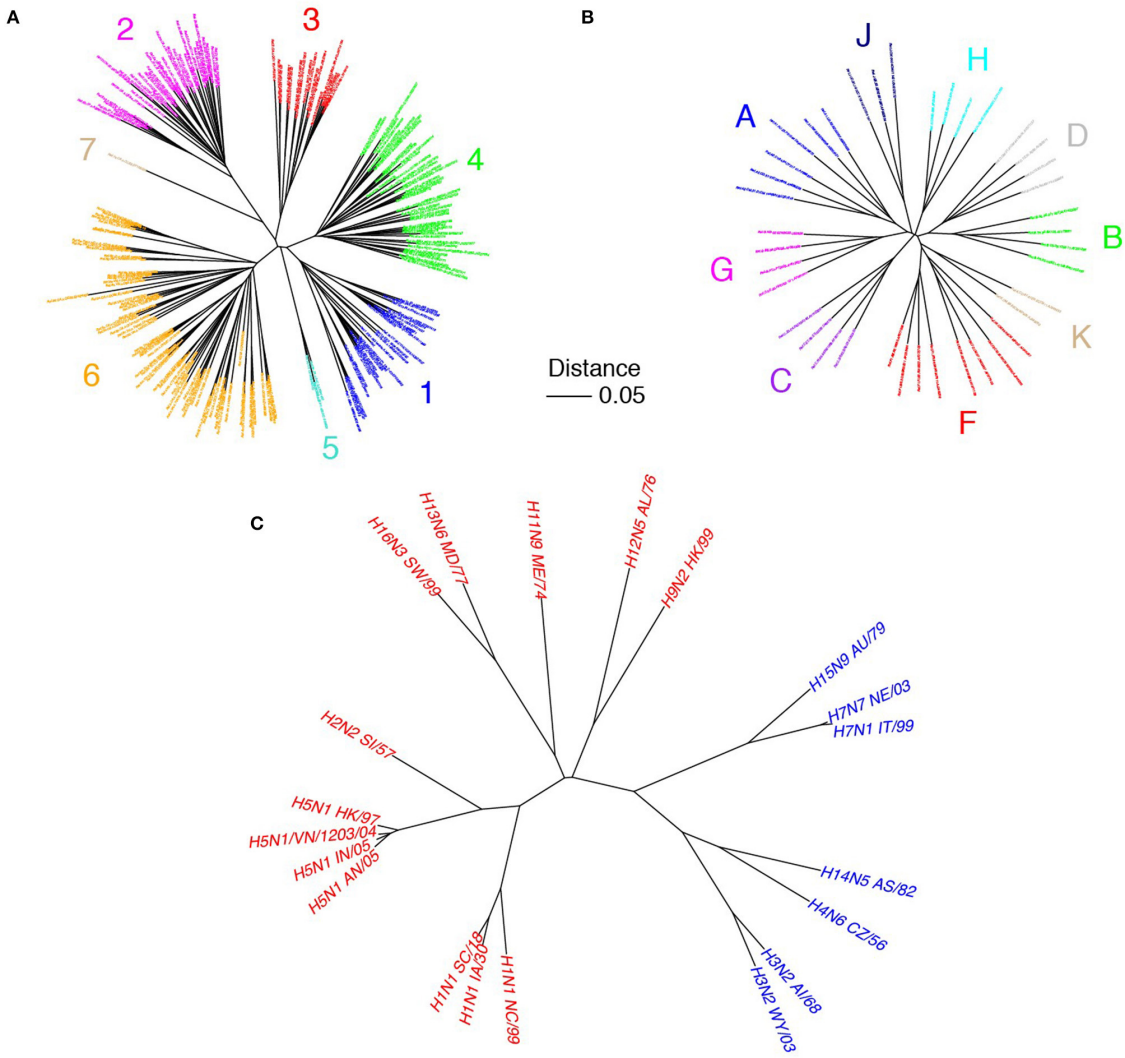
Sequence variability of HCV in comparison with other viruses. Phylogenetic trees of HCV E1E2 **(A)**, HIV env (gp41/gp120; **B**), and influenza hemagglutinin (HA; **C**) are shown. E1E2 amino acid reference sequences (*N* = 204) were downloaded from the LANL HCV database (Kuiken et al., 2005), HIV env reference sequences (*N* = 39) were downloaded from the LANL HIV database (http://www.hiv.lanl.gov), and influenza A HA clones were from Corti et al. (2011), with amino acid sequences downloaded from the Influenza Research Database (Zhang et al., 2017). Multiple sequence alignments were performed using MAFFT software (Katoh and Standley, 2013). Phylogenetic trees were built using the neighbor joining method, and visualized using the APE package (Paradis et al., 2004) in R. Sequence names are labeled, and are colored according to HCV genotype **(A)**, HIV subtype **(B)**, and influenza group (**C**; red = group 1, blue = group 2). Scale bar represents 5% sequence divergence.

The genetic diversity of HCV is high and commensurate with other antigenically variable viruses. Based on alignments of amino acid reference sequences (represented as phylogenetic trees in Figure 1), HCV shows 23% median sequence divergence within genotypes and 33% divergence across genotypes. In contrast, HIV gp120/gp41 (env) has moderately lower sequence variability with 21% median divergence within subtypes and 28% across subtypes, while influenza A virus hemagglutinin has lower variability within hemagglutinin subtype (5%), and much higher sequence variability across subtypes (58%). While specific percent divergence values can vary somewhat based on composition of reference sets, there are clear distinctions in overall glycoprotein sequence diversity among these viruses. Within an infected host, HCV actively avoids immune surveillance (von Hahn et al., 2007) and evolves into a large number of quasispecies through error-prone replication (Tarr et al., 2015) that have up to 10% sequence variations (Simmonds et al., 2005). The lack of proofreading capacity by the viral polymerase leads to a mutational rate of 10^−5^-10^−4^ nucleotides per replication cycle that is a magnitude higher than that for HIV and HBV (Duffy et al., 2008).

Critical to the development of an effective vaccine is the identification and characterization of conserved epitopes associated with viral neutralization, particularly in the E1 and E2 glycoproteins that are the primary neutralizing antibody (nAb) targets (Ball et al., 2014). The E1 and E2 glycoproteins form a heterodimer (E1E2) (Vieyres et al., 2014), and recent evidence suggests that E1 forms trimers on the virion, mediated by the E1 transmembane region, resulting in higher order assemblies containing three E1E2 heterodimers (Falson et al., 2015). These glycoproteins are associated with viral entry via interactions with several cellular receptors, including scavenger receptor class B type 1 (SR-B1) (Scarselli et al., 2002; Fauvelle et al., 2016) and the tetraspanin CD81 (Pileri et al., 1998), as well as fusion with the endosomal membrane once the virus has been internalized by clathrin-mediated endocytosis (Lindenbach and Rice, 2013). The underlying genetic variability occurs despite the requirement for essential interactions between the envelope glycoproteins and cellular receptors necessary for viral entry, and such interactions have been mapped to highly conserved residues in the E2 protein (Drummer et al., 2006; Owsianka et al., 2006; Grove et al., 2008; Rothwangl et al., 2008).

**Figure 2:**
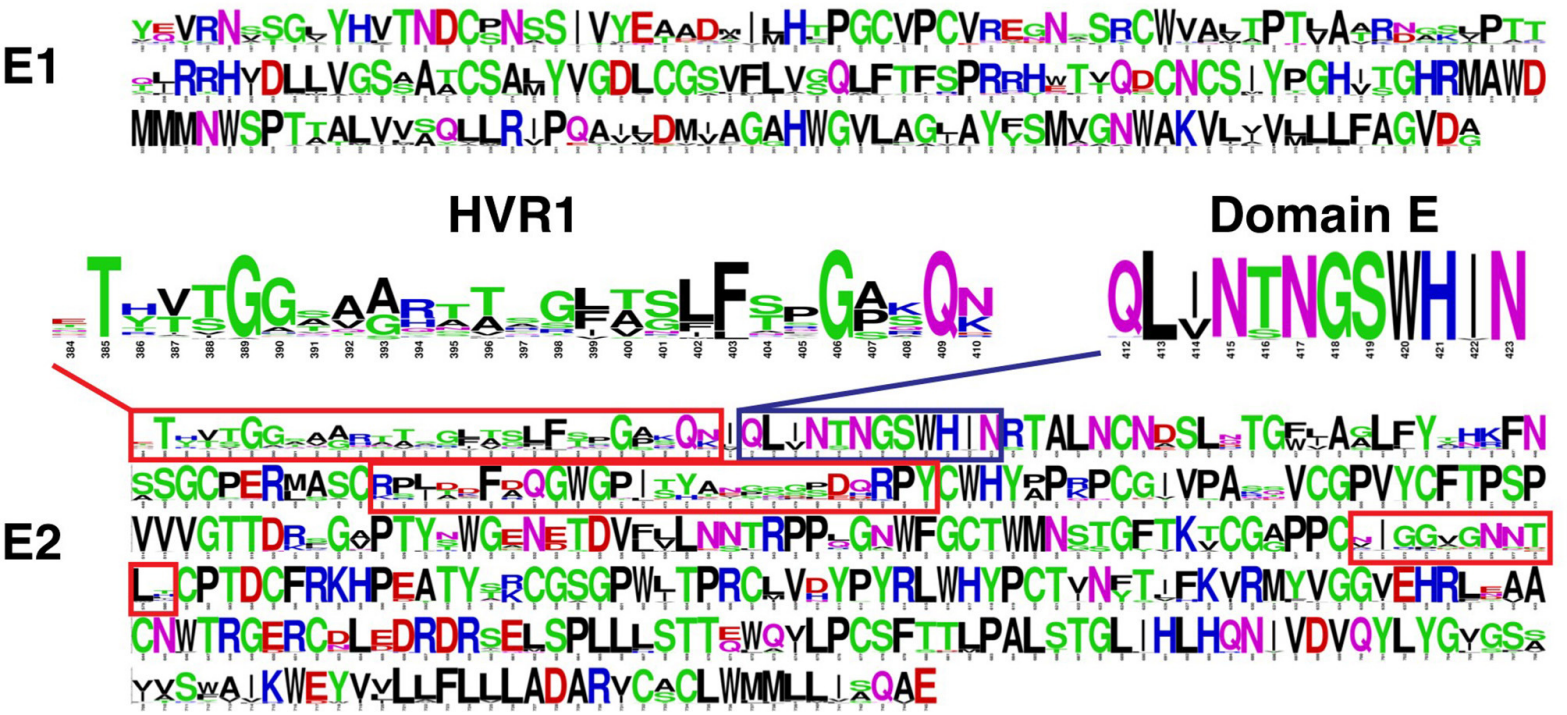
Amino acid sequence variability of HCV envelope glycoproteins E1 and E2 Sequence logos (Crooks et al., 2004) were generated using a multiple sequence alignment of approximately 400 complete E1E2 amino acid sequences downloaded from the Los Alamos HCV database (Kuiken et al., 2005). This gives amino acid propensities at each E1 and E2 position (residues 192-383 and 384-746, respectively, based on the H77 isolate numbering), with total height at each position representing sequence conservation (more variable positions have lower height). Hypervariable regions of E2 are shown in red boxes, and hypervariable region 1 (HVR1; aa 384-410) is highlighted. Antigenic domain E (aa 412-423) is shown in blue box and highlighted. Figure adapted from Pierce et al. (2016a).

The sequence variability of E1 and E2 is not uniform within the protein coding regions (Pierce et al., 2016a). As shown in Figure 2, E1 and E2 include pronounced regions of high amino acid conservation, as well as other regions with considerable sequence variability; the latter category includes hypervariable region 1 (HVR1, aa 384-410), hypervariable region 2 (HVR2, aa 460-485), and intergenotypic variable region (igVR, aa 570-580) on E2. HVR1 (highlighted in Figure 2) is located at the N-terminus of E2 and is under constant immunological pressure. HVR1 serves as a major “immunologic decoy” of the virus (Weiner et al., 1992; Dowd et al., 2009). Other regions of E2 exhibit moderate to complete sequence conservation such as residues 412-423 (antigenic domain E, highlighted in Figure 2) which contains linear epitopes targeted by well-characterized broadly nAbs (Owsianka et al., 2005; Broering et al., 2009; Keck et al., 2013), and residues 441-443 and 523-535 which have been reported to be important for recognition of host entry receptors and broadly neutralizing antibodies (Keck et al., 2008, 2012).

## Mapping antigenic determinants of broad virus neutralization

Cross-competition analyses and epitope mapping of broadly neutralizing human monoclonal antibodies (HMAbs) derived from B cells of subjects with chronic HCV infections have identified at least seven clusters of overlapping epitopes on HCV E1E2. Four clusters, designated as antigenic domains A-D, are composed of conformational epitopes on E2 (Table 1) (Keck et al., 2012). Two additional clusters, designated as antigenic regions (AR) 4 and 5, are composed of conformational epitopes on E1E2 (Kong et al., 2012); representative antibodies AR4A and AR5A had binding determinants mapped to residues in both E1 and E2 using alanine scanning, and did not engage soluble E2 or denatured E1E2 (Kong et al., 2012). It should be noted that the major antigenic region on E2, designated as AR3, overlaps substantially with antigenic domain B by cross-competition and epitope mapping studies (Law et al., 2008). Interestingly, AR5 overlaps with an antigenic domain C HMAb, CBH-7, by competition analysis and epitope mapping, although CBH-7 binds to E2 and does not require E1 (Giang et al., 2012). A seventh cluster of broadly neutralizing antibodies contains overlapping linear epitopes that are located adjacent to HVR1 on E2. This cluster is designated as antigenic domain E and encompasses amino acids 412-423 (Keck et al., 2014). In addition, there are several sites on E1 alone that have been identified as epitopes of neutralizing monoclonal antibodies, including the E1 N-terminus (aa 192-202) which is targeted by antibody H-111 (Keck et al., 2004), and a separate site (aa 313-328) targeted by HMAbs IGH505 and IGH526 (Meunier et al., 2008), the latter of which was characterized structurally in complex with its E1 epitope (Kong et al., 2015b).

**Table 1:** E2 antigenic domains, representative monoclonal antibodies, and representative structures of antibody-epitope complexes in the Protein Data Bank (PDB) (Rose et al., 2011).

**Antigenic domain**	**Residues**	**Antibodies**	**PDB structures**
HVR1	384-410	H77.16	–
A	581-584, 627-633	CBH-4D, CBH-4G, CBH-20	–
B	431-439, 529-535	HC-1, HC-11, CBH-5, AR3C	4MWF (Kong et al., 2013)
C	544-549	CBH-7, CBH-23	–
D	420-428, 441-443	HC84.1, HC84.26	4JZN (Krey et al., 2013), 4JZO (Krey et al., 2013)
E	412-423	HCV1, AP33, HC33.1	4DGY (Kong et al., 2012), 4GAJ (Potter et al., 2012), 4XVJ (Li et al., 2015)

**Figure 3:**
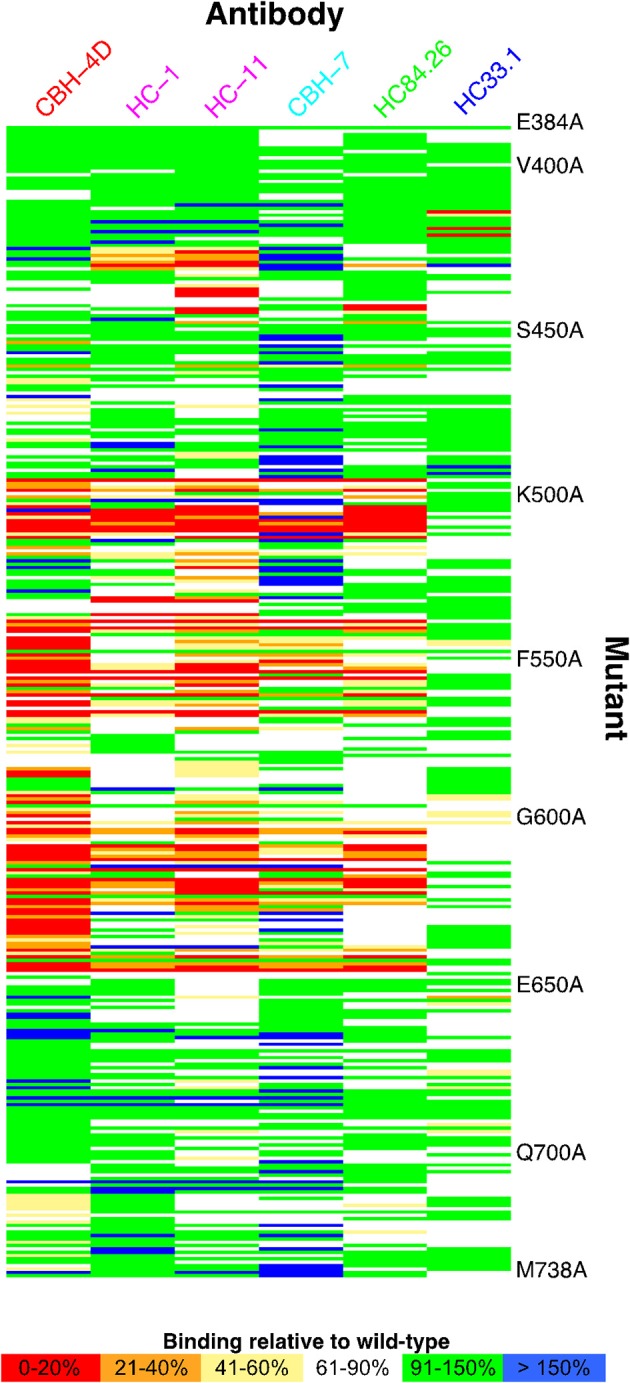
Global E2 alanine scanning, shown as heat map. E2 alanine mutants are on the vertical axis, while HMAbs are on the horizontal axis, colored by antigenic domain (domain A, red; domain B, magenta; domain C, cyan; domain D, green; domain E, blue). Heat map colors represent measured affinity compared with wild-type (H77) E2 as detailed in the legend. Figure adapted from Pierce et al. (2016b).

**Figure 4:**
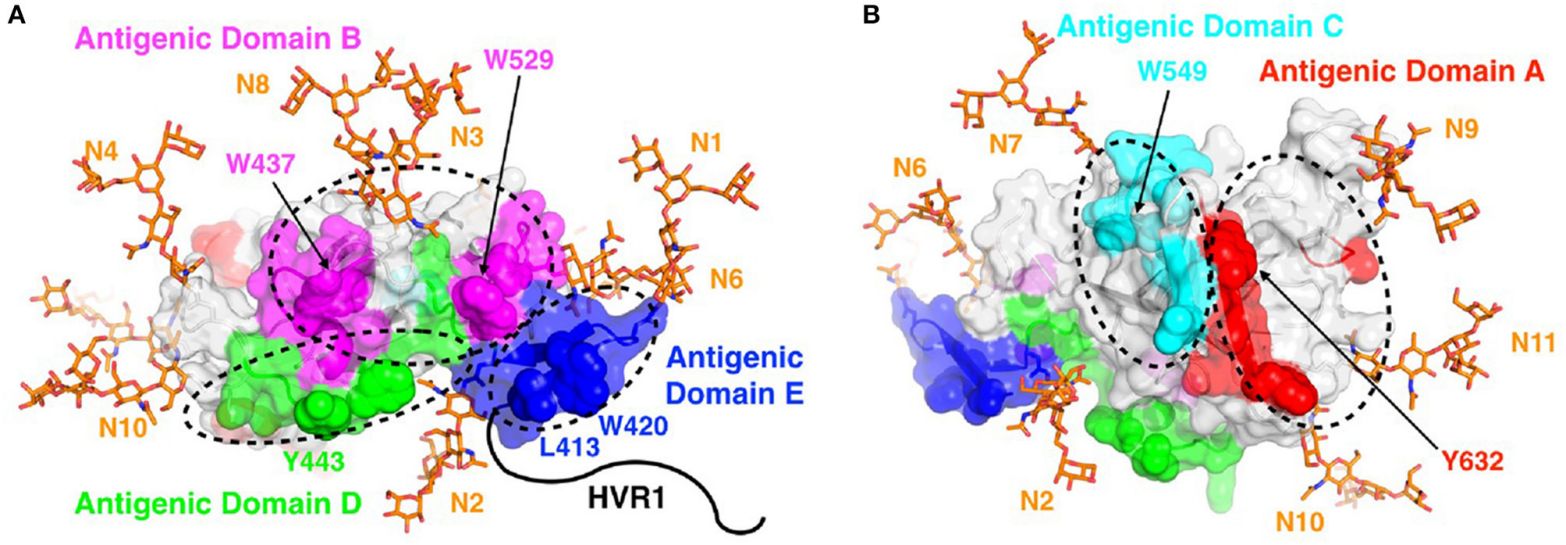
Antigenic domains mapped to the surface of E2. Antigenic domains are colored and labeled on the neutralizing (front layer; **A**) and non-neutralizing (back layer; **B**) face of the E2 core structure (PDB code 4MWF), with key epitope residues shown in space-fill and a subset of those residues labeled. Basic N-glycans were modeled at all 10 sites present in the E2 core structure (N1-N4, N6-N11) using the GlyProt web server (Bohne-Lang and von der Lieth, 2005), and are shown as orange sticks. As the E2 core structure does not include coordinates for the majority of domain E (residues 412-423; of which 412-420 are not present), the domain E coordinates from the domain E-HCV1 complex structure (PDB code 4DGY) were modeled at that site. Hypervariable region 1 (HVR1), which is located at the N-terminus of E2 and also is not represented in the E2 core structure, is shown as a black line for reference. Figure adapted from Fauvelle et al. (2016).

Global alanine scanning of E2 has recently been reported using E2-binding HMAbs and site-directed mutagenesis of the E1E2 coding sequence, and ELISA readout assays to compare mutant binding levels to wild-type (Figure 3; Pierce et al., 2016b). This analysis provided many new insights into the E2 3D structure and determinants of antibody recognition. Although some key binding residues were located on the E2 surface, a large portion of them were buried in E2 core structures (Kong et al., 2013; Khan et al., 2014), including cysteines in disulfide bonds and large hydrophobic residues. These residues are not likely to contact the antibodies directly but rather influence recognition through effects on E2 local or global stability. Computational alanine scanning using the E2 core crystal structure (Kong et al., 2013) to predict E2 stability determinants largely agreed with key E2 sites from global alanine scanning (Pierce et al., 2016b), and also highlighted several positions in a dynamic region targeted by neutralizing antibodies and CD81. Correlations between residues from global alanine scanning were used to predict possible contacts between E2 residues in the native structure; these predictions may be useful in computational modeling of the full E2 and E1E2 glycoprotein structures (recent studies in this regard are noted below). Epitope mapping by alanine substitution studies identified two highly conserved E2 residues Gly530 and Asp535 that are required for binding by all antigenic domain B HMAbs (Keck et al., 2008). Additional residues were identified, Gly523 and Trp529, that are also required for binding by some, but not all, of these antibodies. Importantly, Trp529, Gly530, and Asp535 were previously reported to participate in the interaction of E2 with CD81 (Owsianka et al., 2006). Thus, the broad neutralizing activity of antigenic domain B HMAbs is mediated by competing with CD81 for binding to conserved residues on E2 that are necessary for virus entry. Similarly, three highly conserved residues form a core region for antigenic domain D epitopes at Leu441, Phe442, and Tyr443 (Keck et al., 2012). Leu441 and Tyr443 are absolutely conserved among all known HCV isolates. Phe442, however, is only 60% conserved. Similar to domain B, the domain D region is also involved in interaction with CD81. For AR4- and AR5-specific epitopes that require association of the E1E2 heterodimer, antibodies binding to this region do not mediate neutralization by inhibiting E2 interaction with CD81, but rather potentially block E1E2 engagement with another co-receptor and/or prevent the necessary conformational change in the E1E2 structure required for virus entry (Giang et al., 2012). With the elucidation of the E2 core structure (Kong et al., 2013), the vast majority of these antibodies, antigenic domain B and D, and AR3, are located on the neutralizing face (Figure 4).

Not all broadly neutralizing antibodies share the same anti-viral characteristics. In virus co-culture studies, some antigenic domain B antibodies are associated with viral escape, with and without compromising viral fitness, and others are resistant to viral escape. For instance, at a critical antibody concentration, HC-1, a domain B HMAb, can eliminate infectious virions in cell culture with no viral escape mutants detected. Other antibodies, such as CBH-2 and HC-11 that target the same antigenic domain, permitted viral escape through specific mutations in a region of domain B after repeated passages in the presence of increasing concentrations of the antibodies (Keck et al., 2011). Antibodies to antigenic domain E (aa 412-423) are also associated with different patterns of viral escape. Escape from virus neutralization with AP33 and HCV1 nAbs occurs when there is an N-glycan shift from Asn417 to Asn415 (Chung et al., 2013). This glycan shift, however, does not affect neutralization by other antigenic domain E HMAbs, such as HC33.1 (Keck et al., 2014). Interestingly, escape from antigenic domain D HMAbs has not been observed in co-culture studies even though a critical residue, Phe442, is only 60% conserved (Pierce et al., 2016b). Structural studies provided an explanation of the lack of viral escape in that three residues, located at 441-443, form a hydrophobic protrusion that serves as the binding site for domain D HMAbs (Pierce et al., 2016b). When there is a mutation from F442I or F442L, the interaction with the paratope formed by the heavy chain CDRs leads to a decrease in binding energy of the complex that can be compensated by increasing the antibody concentrations. Taken together, functional and biochemical characterization of broadly reactive nAbs creates a high-resolution database and functional map of neutralizing epitopes that can be strategically used in rational vaccine design.

## HCV envelope structure and antibody recognition

As noted previously, the E1 and E2 glycoproteins form a heterodimer (Op De Beeck et al., 2000), though E2 is the primary, albeit not the exclusive, target of the antibody response. This suggests that E2 is more exposed than E1 in the context of the virion. E2 is heavily glycosylated, typically containing 11 N-glycans at specific sites, while E1 has up to 5 N-glycans (Vieyres et al., 2014). These N-glycans have been characterized using mass spectroscopy (Iacob et al., 2008), and a number of studies have shown that glycans modulate antibody recognition of E2 *in vitro* (Falkowska et al., 2007; Helle et al., 2007) and *in vivo* (Li et al., 2016; Ren et al., 2016). Notably, one E2 N-glycan (N3; position Asn 430) interacts directly with a broadly neutralizing antibody (AR3C), albeit in the interface periphery, in the crystallographic structure of the AR3C-E2 complex (Kong et al., 2013). Additionally, there are four predicted O-glycans on E2, two of which are located in HVR1 (Bräutigam et al., 2013).

Over the past 5 years, a number of studies have helped to elucidate structural features of E1 and E2, as noted in recent reviews (Khan et al., 2015; Kong et al., 2015a; Pierce et al., 2016a). These have collectively shown that key epitopes targeted by broadly neutralizing antibodies on the “front layer” of E2, which corresponds to the CD81 binding face, exhibit structural heterogeneity, in particular E2 antigenic domain E (Kong et al., 2012; Li et al., 2015; Meola et al., 2015), as well as antigenic domains B and D (Kong et al., 2013; Deng et al., 2014; Keck et al., 2016b; Vasiliauskaite et al., 2017). This has been underscored by a recent study, which combined experimental structural and biophysical characterization with simulations to characterize mobility of the CD81 binding region of E2 (Kong et al., 2016).

Two independently determined structures of the E2 glycoprotein “core” region, corresponding to truncations of glycoprotein ectodomains (Kong et al., 2013; Khan et al., 2014), provide major insights into the tertiary structure of E2. Despite the use of distinct monoclonal antibodies, engineered truncations, expression systems, and represented genotypes (1a and 2a), these structures are highly similar overall (approximately 0.8 Å root mean square distance between Cα residues), revealing a globular fold stabilized by numerous disulfide bonds. However, as noted by others in separate reviews (Castelli et al., 2014; Khan et al., 2015), a number of questions remain largely due to (1) discrepancy between disulfide bonds between the current E2 core crystal structures, (2) large critical regions of E2 absent from these structures due to disordered residues or truncation, including HVR1 (aa 384-410), domain E (aa 412-423), HVR2 and flanking residues (aa 453-485), and the ectodomain C-terminal region (aa 646-717), and (3) absence of the E1 portion of the heterodimer. The two studies describing the E2 core crystal structures did include electron microscopy (Kong et al., 2013) and small angle x-ray scattering (Khan et al., 2014) characterization of the larger E2 ectodomains, but their limited resolution does not permit a view of residue interactions, surface residues, or secondary structures, which collectively would be of potential interest for rational vaccine design.

Characterization of the E1 structure has been limited to nuclear magnetic resonance (NMR) structures of short helical regions (Op De Beeck et al., 2000; Spadaccini et al., 2010), an antibody-epitope complex (Kong et al., 2015b), and the N-terminal region of E1, which displayed an unexpected dimeric assembly (El Omari et al., 2014); follow up studies on the latter would help to confirm its representation of the structure of native E1 on the viral envelope. While the above studies have provided key details on the structure and dynamics of key sites, high-resolution structural determination of uncharacterized regions of E1, E2, and, in particular, the E1E2 heterodimer, will be greatly informative for rational vaccine design efforts and understanding of viral assembly.

## Computational modeling of E1E2 assembly

Due to the absence of experimentally determined structures of the E1E2 heterodimer, two recent studies have used computational modeling methods to predict the structure of this assembly (Castelli et al., 2017; Freedman et al., 2017). These models were generated with distinct methods, specifically residue co-evolution analysis based on E1E2 sequence alignments (referred to as evolutionary coupling) to infer residue contacts and guide modeling in one study (Castelli et al., 2017), and a combination of template-based modeling, folding and docking in the other (Freedman et al., 2017). The latter work also employed constraints based on experimental data to guide modeling, and included a model of the transmembrane regions and the putative trimeric assembly of E1E2. As neither of these studies published coordinates of their models, it is not possible to compare directly the extent of convergence between their predicted structures. Both of these studies cross-reference their respective E1E2 structural models with measured alanine scanning and epitope mapping data to provide support, though validation of these models using, for example, experimental characterization of previously uncharacterized mutants of residues predicted to be determinants of E1E2 stability, would provide more conclusive model confirmation. In fact, one of these studies did present pairs of key predicted E1 and E2 interacting residues (Freedman et al., 2017), though no new mutants were tested experimentally in either study. Cross-validation against experimental findings from additional studies, for example recent identification of E1 residues W239, I262, and D263 as putative interface residues with E2 (Haddad et al., 2017), would also help to validate and refine such models.

## HCV host immune evasion strategies

Mechanisms of HCV evasion from the immune system have been described in several recent reviews (Cashman et al., 2014; Dunlop et al., 2015; Pierce et al., 2016a). Extreme levels of viral sequence variability, leading to millions of quasispecies within infected individuals, permit the virus to escape antibody and T cell responses through disruption of immune molecular recognition. A salient example of this mechanism was observed during clinical trials of the HCV1 monoclonal antibody in humans, where initial viral suppression was followed by rebound where isolates exhibited specific amino acid variants at residues N415 and N417 (the latter resulting a shift in glycosylation from residue 417 to residue 415). These variants severely disrupted the ability of HCV1 to bind E2 and neutralize virus, and were rare or undetectable prior to treatment and in patients receiving placebo (Chung et al., 2013; Babcock et al., 2014). Other viral escape mechanisms include epitope shielding by viral glycans, and the presence of hypervariable “decoy” epitopes that elicit antibodies that compete with broadly neutralizing antibodies (Keck et al., 2016a) are summarized in reviews as noted previously.

Recent studies have highlighted the role of E2 co-receptor recognition in the evasion of the antibody response. Using an analysis of monoclonal antibody resistance among a large set of genotype 1 isolates, El-Diwany et al. identified key sites on E2 that permitted escape through modulation of E2 binding to SR-B1 (El-Diwany et al., 2017). Another study found that a set of growth-adapted mutants, isolated from a large library of genotype 2 clones based on the JFH-1 isolate, exhibited increased neutralization by monoclonal antibodies and lower dependence on SR-B1 binding for infectivity (Zuiani et al., 2016). These findings possibly correlate to the observation of a correlation of viral infectivity with overall neutralizing antibody resistance (Urbanowicz et al., 2015).

## Rational design of an HCV vaccine

Approaches for HCV vaccine development have included production of HCV E2 and E1E2 recombinant envelope proteins and use of immuno-adjuvant systems to complex engineering of viral vectors expressing multiple antigens. The rationale for these approaches has been driven by a requirement to elicit multiple broadly neutralizing antibodies to achieve sterilizing immunity to prevent HCV infection and how best to achieve such immunity. Extensive human and animal studies have been performed with a recombinant genotype 1a E1E2 vaccine (Frey et al., 2010; Ray et al., 2010; Wong et al., 2014; Logan et al., 2017). Houghton and his colleagues showed that recombinant E1E2 proteins adjuvanted with an oil-in-water emulsion (MF59C) is safe in humans (Frey et al., 2010) and elicits broadly neutralizing antibodies in both humans and animals (Ray et al., 2010; Wong et al., 2014), as defined by competition analyses of immunized sera from goats and mice against well-established broadly neutralizing MAbs (Wong et al., 2014; Logan et al., 2017). The problem is that a substantial portion of the human antibody responses to this vaccine is directed at the hypervariable region-1 on E2 (Ray et al., 2010) and these antibodies, while neutralizing, are isolate-specific. Taken together, a design approach to direct the antibody responses against conserved epitopes mediating virus neutralization will be advantageous.

The correlation of host antibody response to HCV with viral clearance and recent successes in structure-based vaccine design for other viruses, such as HIV (Jardine et al., 2013; Correia et al., 2014), RSV (McLellan et al., 2013), and influenza (Yassine et al., 2015), suggest that a rationally designed vaccine that elicits neutralizing antibody responses to conserved epitopes is a viable route to an effective B cell based HCV vaccine. Recently, several engineered E1, E2, and E1E2 antigens have been described, including structure-based epitope designs (He et al., 2015; Sandomenico et al., 2016; Pierce et al., 2017). Although the conditions differed for assessing relative immunogenicity in mice, further studies can define the correlates of the magnitude of responses and breadth of protection by neutralization assays for epitope-based and full-length protein-based antigens with designs to minimize viral escape while exploiting the potential Achilles heel of conserved envelope residues. The recent development of an immune competent mouse model with an HCV-related hepacivirus presents a possible means to evaluate and compare such vaccine design strategies (Billerbeck et al., 2017).

Given recent evidence of mobility of key E2 epitopes associated targeted by neutralizing antibodies (Deng et al., 2014; Li et al., 2015; Meola et al., 2015; Kong et al., 2016; Vasiliauskaite et al., 2017), as noted above, stabilization of these epitopes is an intriguing option for future vaccine design efforts, particularly in light of promising recent studies in this regard for other viruses. Recent studies on engineering HIV SOSIP gp140 trimers include designing a stabilized closed protein conformation while decreasing exposure of non-neutralizing epitopes (de Taeye et al., 2015), as well as generation of new “hyperstable” SOSIP designs with engineered disulfide bonds that elicit improved neutralizing antibodies (Torrents de la Peña et al., 2017). Stabilized RSV F immunogens were recently redesigned to further optimize their stability through iterative structure-based design and experimental biophysical and immunological characterization, yielding next-generation RSV immunogens with markedly improved capacity to induce neutralizing antibodies vs. the original designs (Joyce et al., 2016). In that work, the authors noted that their structure-based vaccine design paradigm to optimize antigenic structure and stability “may have utility in the optimization of other vaccine antigens”; HCV E2 would be one promising target in this regard.

## Conclusions

The medical burden of hepatitis C has decreased by the introduction of effective antiviral therapies. However, control of this insidious disease will require the successful development of an effective preventative vaccine. To achieve this goal, the complex interplay between virus and host during acute infection that leads to spontaneous clearance needs to be better understood to provide clues of protective immunity. The criteria for what constitutes a successful immune response leading to viral clearance in designing at vaccine that is broadly cross protective across the genotypes and long lasting will be the holy grail of such vaccine development efforts. Recent insights from deep sequencing, antibody-antigen interactions, structural biology, and immunogenicity studies provide the basis for novel vaccine designs for this challenging target. While accounting for the primary mechanisms of viral escape, namely sequence variability within the viral envelope glycoproteins including prominent decoy epitopes such as HVR1, immunogens can be designed to enhance their capacity to induce potent neutralizing antibodies to conserved epitopes on the viral envelope. These approaches, combined with novel adjuvant formulations capable of eliciting robust and long-lasting humoral and cellular responses, will significantly advance vaccine development efforts to successfully address this daunting medical challenge.

## Author contributions

All authors listed have made a substantial, direct and intellectual contribution to the work, and approved it for publication.

### Conflict of interest statement

The authors declare that the research was conducted in the absence of any commercial or financial relationships that could be construed as a potential conflict of interest.
